# A family of anti-Bacteroidales peptide toxins wide-spread in the human gut microbiota

**DOI:** 10.1038/s41467-019-11494-1

**Published:** 2019-08-01

**Authors:** Michael J. Coyne, Nathalie Béchon, Leigh M. Matano, Valentina Laclare McEneany, Maria Chatzidaki-Livanis, Laurie E. Comstock

**Affiliations:** 1 Division of Infectious Diseases, Brigham and Women’s Hospital, Harvard Medical School, Boston, MA 02115 USA; 2 Institut Pasteur, Genetics of Biofilms Unit, 75015 Paris, cedex 15, 25-28 rue du Docteur Roux, France; 3 Ecole Doctorale Bio Sorbonne Paris Cité (BioSPC), Paris Diderot University, 75013, Cellule Pasteur, Paris, cedex, France; 4 Present Address: Department of Biological Sciences, University of Ohio, Athens, OH 45701 USA

**Keywords:** Microbiome, Bacterial toxins, Metagenomics, Antibiotics

## Abstract

Bacteria often produce antimicrobial toxins to compete in microbial communities. Here we identify a family of broad-spectrum peptide toxins, named bacteroidetocins, produced by Bacteroidetes species. We study this toxin family using phenotypic, mutational, bioinformatic, and human metagenomic analyses. Bacteroidetocins are related to class IIa bacteriocins of Gram-positive bacteria and kill members of the Bacteroidetes phylum, including *Bacteroides*, *Parabacteroides*, and *Prevotella* gut species, as well as pathogenic *Prevotella* species. The bacteroidetocin biosynthesis genes are found in horizontally acquired mobile elements, which likely allow dissemination within the gut microbiota and may explain their wide distribution in human populations. Bacteroidetocins may have potential applications in microbiome engineering and as therapeutics for polymicrobial diseases such as bacterial vaginosis and periodontal disease.

## Abstract (web summary)

Bacteria often produce antimicrobial toxins to compete in microbial communities. Here, the authors identify a family of peptide toxins that are produced by, and target, Bacteroidetes species, some of which are widespread in the human gut microbiota.

## Introduction

Bacteroidetes is a diverse phylum containing several orders of bacteria, some of which are aerobes that live in marine and soil environments, and others, such as the Bacteroidales, which are largely anaerobes. The order Bacteroidales includes several families and genera of bacteria that are typically members of host-associated microbial communities. The best studied Bacteroidales include the *Bacteroides* and *Parabacterioides*, which are abundant members of the healthy human gut microbiota, *Porphyromonas gingivalis*, which is an important periodontal pathogen, and *Prevotella spp*., which occupy diverse ecosystems including the human gut, vagina, oral cavity, cow rumen, as well as environmental sites. Long before the advent of the microbiome era, microbiologists studied several species of Bacteroidales due to their involvement in disease^1–5^.

Bacteroidetes members typically live in complex microbial communities where the production of antibacterial molecules against related members provides a competitive advantage. Compared to some members of the Firmicutes and Proteobacteria phyla where antimicrobial molecules have been studied for decades^6,7^, there are still few studies that have analyzed the production of antimicrobial molecules by the Bacteroidetes. It is only within the last five years that antibacterial toxins have been identified from members of this phylum. Most *Bacteroides fragilis* strains synthesize contact dependent Type VI secretion systems whose toxins kill diverse *Bacteroides* and *Parabacteroides* species^8–11^. Several diffusible antibacterial toxins have also been identified, the most wide-spread being the membrane attack comlex/perforin (MACPF) proteins, for which there are hundreds of proteins encoded by diverse Bacteroidetes genomes. Several MACPF proteins have been shown to have toxin activity and are specific for intra-species killing^12–14^. A secreted eukaryotic-like ubiquitin molecule produced exclusively by a subset of *B. fragilis* strains specifically antagonizes other *B. fragilis* strains^15^.

In this study, we identify a family of peptide toxins produced by diverse members of the Gram-negative Bacteroidetes phylum. We show that these bacteriocin genes are widely disseminated in human gut microbiomes and target a broad-spectrum of diverse Bacteroidetes species including environmental, symbiotic and pathogenic members.

## Results

### Strains producing anti-*Bacteroides*/*Parabacteroides* toxin(s)

**Fig. 1:**
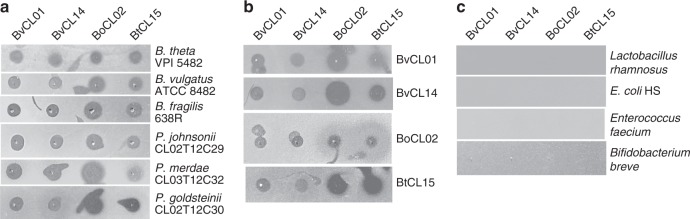
Agar spot overlay assays demonstrating broad-spectrum toxin production by four *Bacteroides* strains. The four toxin-producing strains are shown at the top and were cultured on plates overnight and removed, then the plates were overlaid with top agar containing the strains shown on the left to test for growth inhibition. A dark spot indicates growth inhibition of the overlaid strain. **a** Six *Bacteroides* and *Parabacteroides* strains used in the overlays. **b** The toxin-producing strains used in the overlays. **c** Non-Bacteroidetes gut strains from three different phyla used in the overlays

A screen of 140 different human gut *Bacteroides* and *Parabacteroides* strains of 15 different species revealed that four of the strains produce a secreted molecule that targets all *Bacteroides* and *Parabacteroides* species tested under the conditions of our agar overlay spot assay (Fig. 1a, Supplementary Fig. 1). These four toxin-producing strains include a *Bacteroides ovatus* (BoCL02T12C04), a *Bacteroides thetaiotaomicron* (BtCL15T12C11), and two *Bacteroides vulgatus* strains (BvCL01T12C17 and BvCL14T03C19). As shown in these assays, the secreted toxin that remains on the plate after the producing bacteria are removed often travels along the surface of the plate in irregular patterns when the second strain is added to the plate in the overlay. This phenomenon results in growth inhibition streaks and spots stemming from the original spot and at times distant from the dotted producer strain (Fig. 1, Supplementary Fig. 1). We have not seen this phenomenon in other secreted antibacterial toxins produced by *Bacteroides* species^12–15^ suggesting that these toxins possess different physical properties further supported by the fact that they do not penetrate and diffuse well in the agar. These toxin-producing strains also inhibit their own growth in these assays, suggesting that they do not produce immunity proteins (Fig. 1b).

We also tested a commensal *E. coli* strain (phylum Proteobacteria), a *Lactobacillus rhamnosus* and *Enterococcus faecium* strain (phylum Firmicutes), and a *Bifidobacterium breve* strain (phylum Actinobacteria). None of these gut bacteria were growth inhibited by a molecule secreted by these four strains (Fig. 1c).

### Genetic regions involved in toxin production

**Fig. 2:**
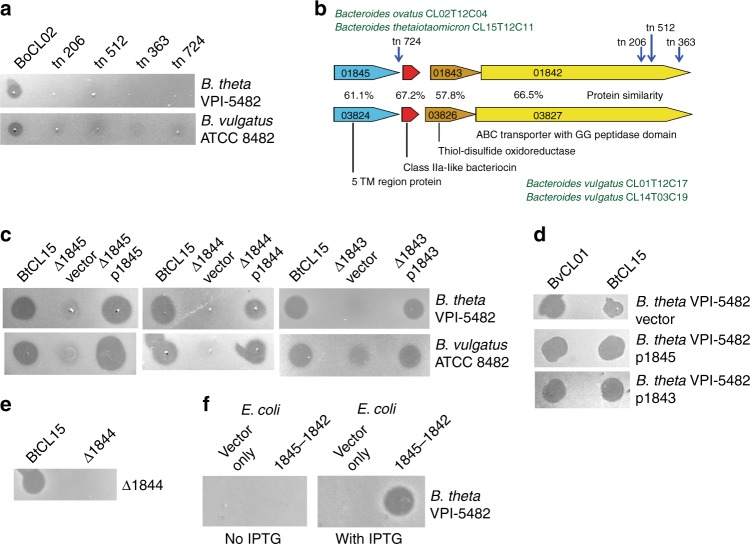
Identification and analysis of bacteriocin biosynthesis regions**. a** Agar overlay assays analyzing growth inhibition activity of BoCL02T12C04 and four transposon mutants against B. *thetaiotaomicron* VPI-5482 and *B. vulgatus* ATCC 8482. **b** ORF maps of a four-gene genetic region common to BoCL02 and BtCL15 showing the insertion sites of the transposon mutants (vertical blue arrows) that abrogated toxin production. Gene names of the BtCL15 strain beginning with the prefix EH213_0 are shown inside each gene. A similar genetic region from the BvCL01 and BvCL14 strains is shown below and the percent similarity of the proteins of these two regions is shown. Putative functions or properties of the proteins encoded by each gene are indicated. **c** Agar overlay assays analyzing the effects of deletion of three genes in the toxin biosynthesis region on the ability of BtCL15 to inhibit the growth of the two sensitive strains. **d** Agar overlay assays testing for the ability of the genes encoding the five transmembrane protein (p1845) or the thiol oxidoreductase (p1843) to protect *B. thetaiotaomicron* VPI-5482 when expressed *in trans*. **e** Agar overlay assay analyzing the ability of wild-type BtCL15 and the putative peptide bacteriocin gene deletion (Δ1844) to inhibit the growth of Δ1844. **f** Agar overlay assay analyzing the production of toxin from *E. coli* expressing the four-gene region (EH213_1845-1842) from an IPTG inducible promoter

To identify the gene(s) involved in toxin production and to determine if the four strains produce the same secreted toxin, random transposon mutagenesis was performed using strain BoCL02T12C04 (BoCL02). Four transposon mutants were identified that abrogated the inhibitory activity using *B. thetaiotaomicron* VPI-5482 as the sensitive strain in the screening assay (Fig. 2a). The four transposon insertions were mapped and found to span an approximately 3-kb region (Fig. 2b). Of the four toxin-producing strains, only the genome sequence of BoCL02 was available; therefore, we sequenced the genomes of the other three strains. DNA-level comparative analysis revealed that BtCL15T12C11 (BtCL15) has a 100% identical region to this 3-kb region of BoCL02 (Fig. 2b) but the two *B. vulgatus* strains do not have a region similar at the DNA level. Analysis of the interrupted genes showed that one encodes a protein similar to an ABC-like bacteriocin transporter with an N-terminal double-glycine peptidase domain. Proteins of this family are involved in removing the leader peptide following a GG motif of class IIa bacteriocins and their subsequent secretion across the membrane of Gram-positive bacteria^16,17^. Indeed, a gene encoding a small protein with characteristics similar to class IIa-like bacteriocins is also included in this region (Fig. 2b)^18^. Similarity searches using tblastn with the proteins encoded by this region revealed similar proteins in the two *B. vulgatus* strains, encoded by genes with the same genetic order (Fig. 2b). The DNA of these two regions in the *B. vulgatus* genomes are 100% identical to each other, and the proteins encoded are 57.8%–67.2% similar to those encoded by the BoCL02/BtCL15 genes (Fig. 2b). Both regions included four genes encoding similar products with the following predicted features/annotations: 1) an unknown protein with five transmembrane regions (TM), 2) a small bacteriocin-like molecule, 3) a thiol oxidoreductase, and 4) an ABC-like bacteriocin transporter with a double-glycine peptidase domain. The products encoded by the genes flanking these four-gene regions are not similar between these two genetic types.

In Gram-positive bacteria, class IIa bacteriocin genetic regions typically include not only the bacteriocin gene and bacteriocin peptidase/transporter, but also a gene encoding an immunity protein. The TM protein and thiol oxidoreductase encoded by the two genes flanking the putative bacteriocin genes are typically not present in class IIa bacteriocin regions. To determine if either the TM or the thiol oxidoreductase genes are necessary for secreted toxin activity, internal non-polar deletion mutants of these two genes and the putative bacteriocin gene were constructed individually in the BtCL15 background strain. Deletion of any of these three genes results in attenuated toxin activity that is restored when the respective gene is added *in trans* (Fig. 2c).

As there are no obvious immunity proteins encoded by these regions, we determined whether either the TM protein or the thiol oxidoreductase may serve that function. Expression of either gene from the BoCL02 strain in *B. thetaiotaomicron* VPI-5482 did not protect the organism from the activity of either the BoCL02/BtCL15 toxin or the BvCL01/BvCL14 toxin (Fig. 2d). In addition, the deletion mutant of the putative bacteriocin peptide gene (Δ1844), which produces the three other proteins of this region based on complementation analyses (Fig. 2c), is still growth inhibited by the BtCL15 wild type strain (Fig. 2e). These findings are not surprising as these toxins also kill their wild-type producing strains in the agar overlay assay, suggesting no self-protection by an immunity protein (Fig. 1b).

The combined data of the transposon mutagenesis, deletion mutant analyses, and comparison of the Bo/Bt and Bv regions suggest that these four genes may be the extent of the toxin biosynthesis region. To determine if these four genes are sufficient to confer toxin activity to a heterologous species, we cloned the four genes into pET16b where expression of the genes is induced by the addition of IPTG, and transformed this plasmid into *E. coli* BL21(DE3). When IPTG was present in the LB plate used to culture the *E. coli*, the strain inhibited the growth of *B. thetaiotaomicron* VPI-5482 (Fig. 2f). Therefore, these combined data show that this four-gene region is sufficient for active toxin production.

### Analysis of the two putative peptide bacteriocins

**Fig. 3:**
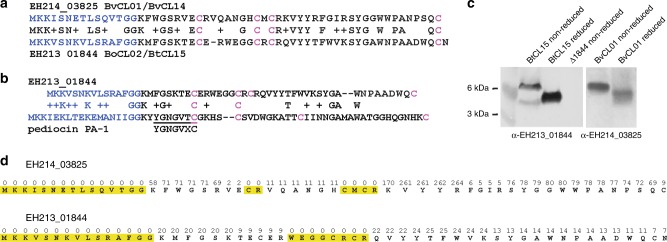
Analysis of the peptides produced from wild-type bacteria. **a** Alignment of the bacteriocin-like peptide of BvCL01/BvCL14 with that of BoCL02/BtCL15. The blue N-terminal sequence is the leader peptide predicted to be cleaved after the GG motif. Four cysteine residues in each peptide are highlighted in pink. **b** Alignment of the BoCL02/BtCL15 peptide with the class IIa bacteriocin pediocin from *Pediococcus spp*.. A conserved motif in class IIa bacteriocins of Gram-positive bacteria is underlined. **c** Western immunoblot analysis of the migration of reduced and non-reduced peptides from BvCL01 or BtCL15 probed with an antiserum generated to the synthesized peptides. **d** Results of LC–MS/MS analysis of the peptides from BvCL01/BvCL14 or BtCL15 showing the number of times each residue was detected by the number above. Yellow highlights indicate regions that were not detected by LC–MS/MS analysis. Source data are provided as a Source Data File

The two putative peptide toxins produced by the regions shown in Fig. 2b are 67.2% similar to each other (Fig. 3a). Both have leader sequences of 15 amino acids that are predicted to be cleaved after the GG motif (Fig. 3a), resulting in mature peptides of 42 amino acids. Each peptide has four cysteine residues that in class IIa bacteriocins are involved in intramolecular disulfide bonds. As *Bacteroides* do not have the general machinery for disulfide bond formation^19^, the thiol oxidoreductases encoded in these regions likely catalyze the formation of disulfide bonds in these peptides. Although having some similar properties to class IIa bacteriocins, the peptides lack the YGNGVXC domain conserved in class IIa bacteriocins^20^ and the mature peptides have very little sequence similarity to class IIa bacteriocins (Fig. 3b). Each of the mature 42 aa peptides has 12–13 hydrophobic amino acids and both mature peptides are also somewhat basic with the *B. vulgatus* peptide having a net charge of +6 and the Bo/Bt peptide a net charge of +2 at pH 7.

To determine if these peptides are cleaved at the GG motif and if they likely form disulfide bonds, we analyzed the peptides produced by wild-type BvCL01/BvCL14 or BtCL15 grown on plates to obtain more concentrated toxin than obtained from broth grown cultures. Each of the peptides was also chemically synthesized without the putative leader sequence and used to generate antibodies to visualize the natural molecules by western immunoblot. Both naturally produced peptides migrate differently under reduced compared to non-reduced conditions (Fig. 3c), consistent with the presence of disulfide bonds^21^. To determine if the leader sequence is cleaved at the double glycine motif as predicted, samples prepared as described above for the western blots were concentrated, reduced, alkylated, run on polyacrylamide gels and stained with Coomassie. Proteins in the molecular mass range indicated by the western blots were excised and subjected to LC–MS/MS analysis. We identified fragments covering 85.7% and 81.0% of the mature peptides, respectively (Fig. 3d, Supplementary Fig. 2). No fragments were detected corresponding to any portion of the leader sequence, whereas 58 and 20 fragments included the lysine residue immediately following the GG site for each peptide (Fig. 3d, Supplementary Fig. 2). For both peptides, fragments corresponding to the portion containing the central cysteine residues were also not detected by this LC–MS/MS analysis (Fig. 3d, Supplementary Fig. 2). Lack of detection of this central portion may be due to the fact that cysteine containing peptides are more difficult to identify due to incomplete reduction and alkylation, especially when two cysteine residues are in close proximity^22^.

### Synthesized peptide confers killing activity

**Fig. 4:**
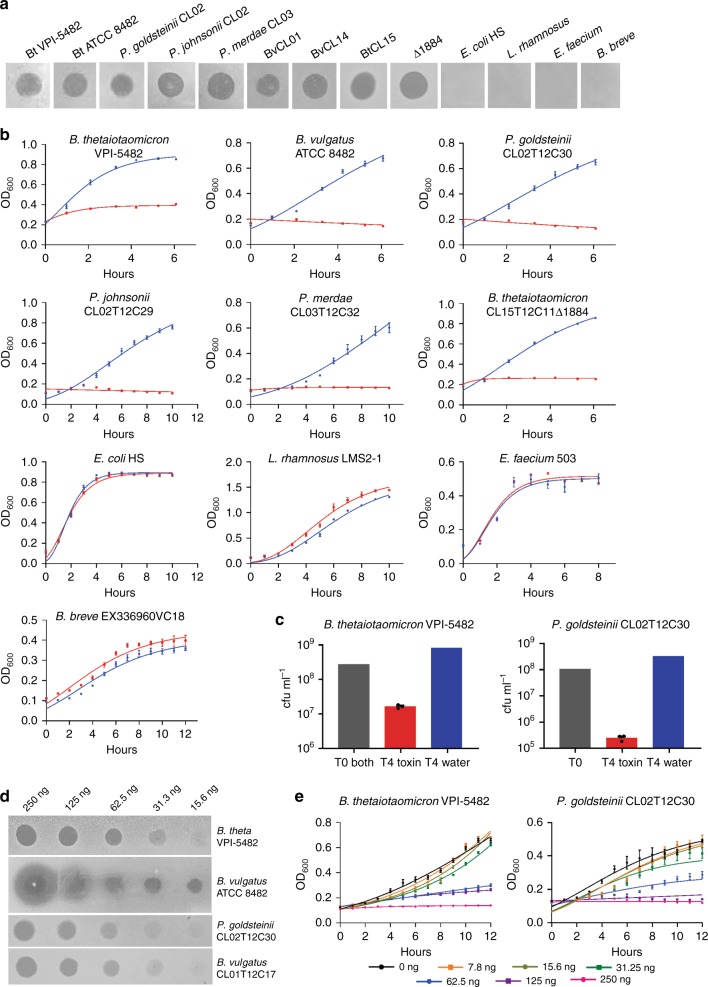
Analysis of killing activity by synthesized 3825 peptide. **a** Growth inhibition assays by the 3825 peptide (bacteroidetocin A) when 1 µg is applied in a 5 µl spot on top of an agar overlay containing the indicated strains. **b** Growth inhibition assays in broth culture when 2 µg of bacteroidetocin A (red) or water control (blue) is added to the indicated strain. All data represent the average of biological triplicates, ±SEM. **c** Colony forming unit (cfu/ml) quantification of viable bacteria following four hours in growth medium with bacteroidetocin A (red) compared to the starting cfu/ml (gray), or after four hours growth with added water (blue). Biological triplicates were performed for the growth assay with added bacteroidetocin A. Error bars represent standard error of the mean. **d** Agar overlay assays demonstrating the amount of bacteroidetocin A necessary to inhibit the growth of the overlay strains. **e** Titration of 3825 peptide in broth to determine the minimal inhibitory concentration for growth inhibition of *B. thetaiotaomicron* VPI 5482 and *P. goldsteinii* CL02. Source data are provided as a Source Data File

To confirm that these small peptides are responsible for the broad-spectrum Bacteroidales growth inhibition, we used the chemically synthesized 42 amino acid peptides representing the mature form lacking the leader sequence. The Bo/Bt peptide is quite insoluble in water and attempts to solubilize it with mild acid were successful, but the acid solvent itself had a growth inhibitory effect (data not shown). The *B. vulgatus* peptide (EH214_03825 (3825) from the BvCL01 strain), is soluble in water and therefore was used for subsequent assays of peptide activity. For class IIa bacteriocins, the concentration of peptide necessary to inhibit growth varies with each peptide and the strain tested for sensitivity^23,24^. We used a quantity of peptide (1 µg of peptide in 5 µl water) where a zone of inhibition should be detected if the strain is sensitive. This peptide has a broad-spectrum inhibitory activity against diverse *Bacteroides* and *Parabacteroides* species, but not against other gut species of distinct phyla (Fig. 4a). We also performed growth assays in broth culture using the synthesized peptide. This molecule inhibited the growth of all strains that were inhibited in the overlay assay, but not gut strains of different phyla (Fig. 4b). To determine if the toxin was killing the bacteria or only arresting growth, both a *Bacteroides* and *Parabacteroides* strain were plated to quantify cfu/ml after four hours of culture in the broth assay with the synthesized 3825 peptide toxin. Both strains had reduced cfu/ml (1–3 log reduction) following culture with toxin when compared to the cfu at the beginning of the incubation (Fig. 4c). In contrast, bacteria incubated with just added water (the toxin solvent) increased in cfu/ml compared to the starting inoculum as expected (Fig. 4c). To determine the potency of this toxin, we quantified the amount of toxin necessary to produce a visible growth inhibition zone in the agar overlay assay and found that as little as 31 ng produced a visible zone against both *Bacteroides* and *Parabacteroides* species (Fig. 4d). We performed similar toxin titration assays in liquid culture and found that as little as 62.5 ng/ml (12.5 nM) was able to inhibit the growth of both the *Bacteroides* and *Parabacteroides* strains tested (Fig. 4e). This concentration is comparable to minimal inhibitory concentration of pediocin PA-1^25^. Based on our demonstration of toxin activity by these peptides, either directly for the BvCL01 toxin 3825, or indirectly by mutational analysis for the BtCL15 toxin (1844), we named these toxins bacteroidetocins A and B, respectively (for Bacteroidetes bacteriocins).

### Bacteroidetocin A inhibits *Prevotella* of diverse ecosystems

**Fig. 5:**
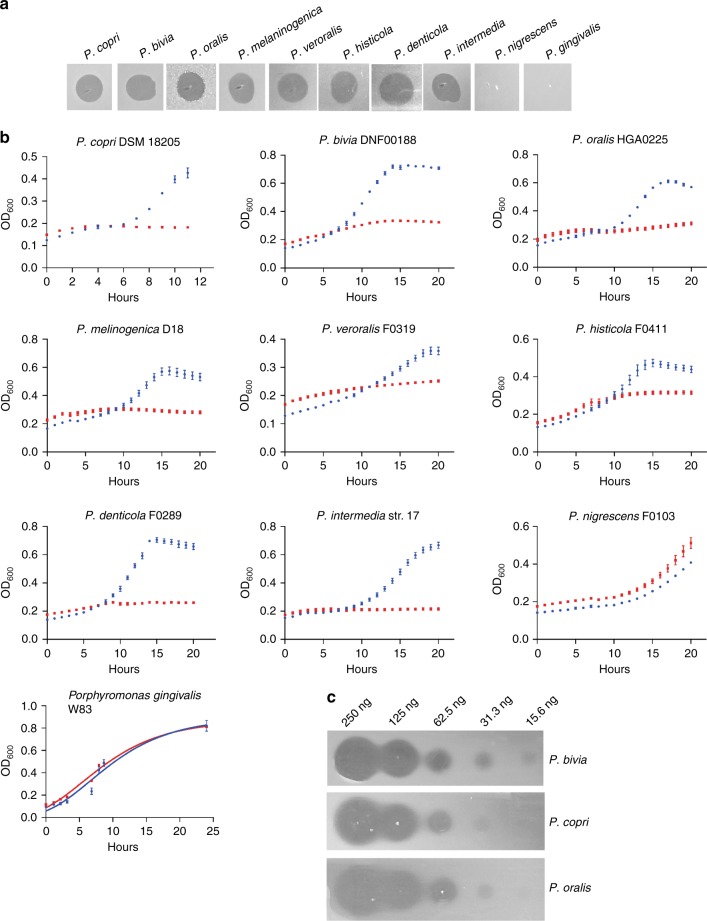
Ability of bacteroidetocin A to inhibit the growth of *Prevotella* strains. **a** Growth inhibition assays by bacteroidetocin A when 1 µg is applied in a 5 µl spot on top of an agar overlay containing the indicated strains. **b** Growth inhibition assays in broth culture when 2 µg of baceroidetocin A (red), or water control (blue) is added to the indicated strain. **c** Agar overlay assays demonstrating the amount of bacteroidetocin A necessary to inhibit the growth of the overlay strains. Source data are provided as a Source Data File

We next analyzed the ability of synthesized bacteroidetocin A to target *Prevotella* species of diverse ecosystems in both the agar overlay assay as well as in liquid culture. These strains included the gut *Prevotella* species *P. copri* and several pathogenic *Prevotella* species of the mouth and of the vagina. In addition, we tested the toxin’s effect on *P. gingivalis*, a major Bacteroidales pathogen of periodontal disease. All Bacteroidales species tested were inhibited by the toxin with the exception of two: *Prevotella nigrescens* and *P. gingivalis* (Fig. 5a, b). The amount of toxin necessary to yield a zone of inhibition of a gut, vaginal, or oral *Prevotella* strain was similar to the *Bacteroides* and *Parabacteroides* strains, where as little as 31 ng yielded a small yet visible zone of inhibition (Fig. 5c). We also tested three species of the Bacteroidetes phylum that are not members of the order Bacteroidales: *Sphingobacterium spiritovorum* and *Sphingobacterium multivorum* (order Sphingobacteriales), and *Elizabethkingii meningcepticum* (order Flavobacteriales), and found they are not affected by the toxin in the agar assay (data not shown).

### Colonization levels in the gnotobiotic mouse gut

**Fig. 6:**
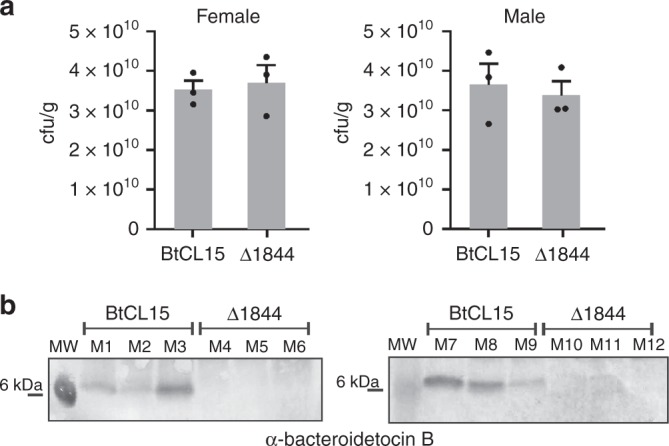
Colonization level of bacteroidetocin B-producing and bacteroidetocin B mutant strain (BtCL15Δ1844) in the gnotobiotic mouse gut. **a** Colonization levels of gnotobiotic mice monoassociated with BtCL15 or BtCL15Δ1844. Fecal samples were collected after 6 days. The mean cfu/ml recovered from groups of three mice of each sex is plotted with the SEM as error bars; *P* value > 0.9999 for both groups of mice. The statistical analysis was performed using a two-tailed Mann–Whitney test as calculated by Prism version 8.0.1 for 64-bit Windows (GraphPad Software, San Diego, CA). **b** Western immunoblots of feces of mice monoassociated with BtCL15 or BtCL15Δ1844 for 6 days using 1844 (bacteroidetocin B) antiserum. M1–M3 and M7–M9 were monoassociated with wild-type BtCL15 and M4–M6 were monoassociated with BtCL15Δ1844. Source data are provided as a Source Data File

The four toxin-producing strains were all previously isolated in our lab as part of a study designed to analyze abundant Bacteroidales species in the feces of a cohort of 15 healthy humans in the Boston area^26^. Therefore, despite the fact that four of these bacteroidetocin A- or B-producing strains self-intoxicate, each colonized these humans at 10^8^ cfu/gram of feces or greater in the complex human gut ecosystem (the lowest concentration that was plated). To determine if toxin production impairs colonization in the mammalian gut, we monoassociated gnotobiotic mice with the BtCL15 wild-type strain or the isogenic toxin gene deletant (Δ1844). Both the wild-type strain and the toxin mutant achieved very high levels of colonization (~3 × 10^10^ cfu/gram of feces) with no significant difference in colonization between the two groups of mice, regardless of sex (*P* value > 0.9999 using a two-tailed Mann–Whitney test) (Fig. 6a). Using the antiserum to bacteroidetocin B, we show that the toxin is present in the stool of mice monoassociated with the wild-type, albeit at different levels (Fig. 6b), using the stool of mice colonized with the isogenic toxin gene deletion as the negative control. Therefore, we did not detect a colonization disadvantage to the wild-type strain due to the production of bacteroidetocin B in this model system.

### Bacteroidetocin regions are part of larger genetic elements

**Fig. 7:**
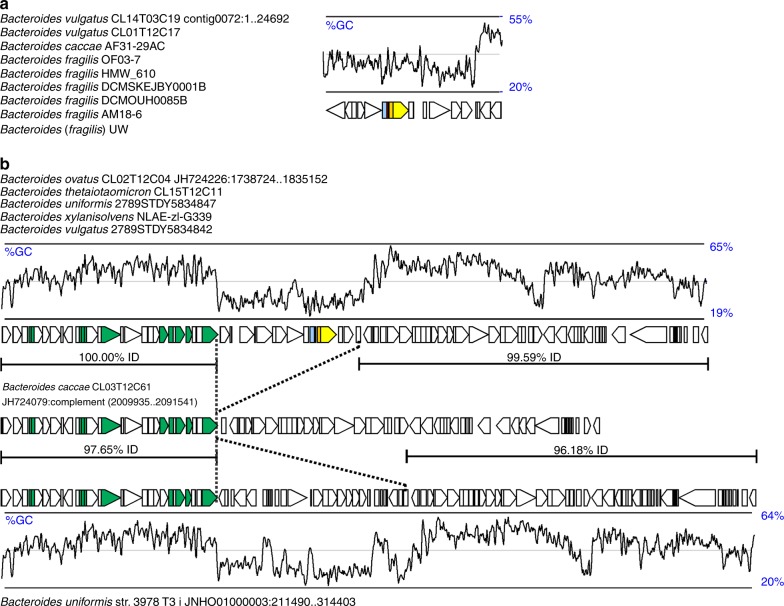
Analysis of the extended genetic regions of the bacteroidetocin A and B biosynthesis genes. **a** Left - Names of all strains in our genome collection with the bacteroidetocin A biosynthesis region. Right - A 26-kb region common to all strains with the bacteroidetocin A biosynthesis genes (colored). A sliding window plot of the G+C content of the region is shown above. **b** Top - Names of all strains in our genome collection with the bacteroidetocin B biosynthesis region. Top orf map - A 96-kb integrative and conjugative element (ICE) common to all strains with the bacteroidetocin B biosynthesis genes (colored). Genes typical of ICE involved in mobility, excision and transfer are colored green. Middle panel – a similar genetic region present in the genome of *B. caccae* CL03T12C61. This region lacks the ~19-kb region present in the bacteroidetocin B ICE. Dotted line delineate the regions of divergence. The extent and percent DNA identity between regions is indicated with horizontal lines. Bottom panel – a similar ICE from strain *B. uniformis* CL03T12C37 that has a distinct ~26-kb central portion. A sliding window plot of the G+C content of the region is shown below

To determine the prevalence of these two regions among sequenced Bacteroidetes/Chlorobi superphylum genomes and the diversity of species that harbor them, we searched for the same genetic regions in other sequenced genomes. Of the more than 600 *Bacteroides* strains with genome sequences in our curated collection, the bacteroidetocin A gene region is present only in seven other strains including *B. caccae* and *B. fragilis* strains (~99% DNA identity of the four gene region between strains) (Fig. 7a). The bacteroidetocin B region was found in three other strains (>99% DNA identity of the four-gene region), each of different species: *B. uniformis*, *B. xylanisolvens*, and *B. vulgatus* (Fig. 7b). Neither of these toxin genetic regions were found in non-*Bacteroides* species.

We analyzed the DNA surrounding these four-gene regions to determine the length of DNA common to all strains with a given bacteroidetocin region. The extent of the conserved DNA between the nine strains with the bacteroidetocin A region is ~25 kb. Although this region is present in strains of three different species, there are no recognizable conjugal transfer genes or other genes to suggest a mechanism of transfer. The average G+C content of the ~25 kb region is ~35%, which is less than the ~43% average of *Bacteroides* genomes.

Analysis of the bacteroidetocin B region demonstrated it is part of a ~96-kb integrative and conjugative element (ICE) (Fig. 7b). This large ICE is greater than 97% identical between the five strains. Obvious transfer genes characteristic of ICEs are present on this element (shown in green). A search of Bacteroidetes genomes for similar ICEs revealed two other variants, each with nearly identical genes but lacking or having a replacement for the interior ~19-kb region containing the bacteroidetocin B biosynthesis region (Fig. 7b). The ICE from *Bacteroides caccae* CL03T12C61 lacks the ~19-kb central region containing the bacteroidetocin B cluster. Another similar ICE was found in 13 other *Bacteroides* strains, including *Bacteroides uniformis* str. 3978 T3 I (Fig. 7b). This ICE also lacks the ~19-kb region containing the bacteroidetocin B locus, and instead includes ~26-kb of DNA that inserted into the same region of the smaller ICE, possibly indicating a recombination hot spot within these ICE. Although there is extensive fluctuation in GC content along the ICE containing the bacteroidetocin B locus, the lowest GC region is the ~19-kb kb region containing the bacteroidetocin B biosynthesis locus (average GC 31.1%). This finding suggests that this bacteriocin region may have originally been acquired from a low G+C bacterium such as a Gram-positive bacterium.

### Prevalence of bacteroidetocin A and B in human gut metagenomes

Although the bacteroidetocin A and B genetic regions are not present in many of the sequenced *Bacteroides* genomes, our finding of *Bacteroides* strains with these toxins in the gut ecosystems of four of our 15 human subjects suggests they may be more prevalent than indicated by the analysis of sequenced genomes. To better understand the distribution of bacteroidetocin A- and B-producing strains in the human population, we analyzed the “3 consortium gene catalog” (3GCG) human gut metagenomic dataset^27^ that includes 1070 human subjects from four different countries. Using tblastn to search for bacteroidetocin A and B protein sequences, we identified many human gut metagenomes with sequences matching bacteroidetocin A and B, most at 100% identity (Table 1, Supplementary Data 1). Bacteroidetocin A is present in ~7.6% of human gut metagenomes with a somewhat even distribution among nationalities (Table 1). Bacteroidetocin B, however, is highly overrepresented in the American population, with nearly one-quarter of metagenomic samples containing this toxin gene. This is a highly significant difference compared to metagenomes from individuals from other countries from which bacteroidetocin B was detected in 1.9–6% of metagenomes (*P* < 0.0001). Bacteroidetocin B is also significantly under-represented in Chinese gut metagenomes (*P* = 0.001) compared to other nationalities. As we only detected bacteroidetocin A and B biosynthesis regions in *Bacteroides* species, it is possible that an uneven prevalence of *Bacteroides* strains in these human populations may account for some of this difference.

**Table 1:** Prevalence of bacteroidetocin A and B in human gut metagenomes

	EH214_03825 (bacteroidetocin A)		EH213_01844 (bacteroidetocin B)		either toxin gene	
nationality (number)	number	%	number	%	number	%
American (94)	10	10.64%	23	24.47%	33	35.11%
Chinese (368)	18	4.89%	7	1.90%	25	6.79%
Danish (401)	38	9.48%	24	5.99%	62	15.46%
Spanish (207)	15	7.25%	4	1.93%	19	9.18%
**Total (1070)**	81	7.57%	58	5.42%	139	12.99%

### Additional bacteroidetocins encoded by Bacteroidetes genomes

**Fig. 8:**
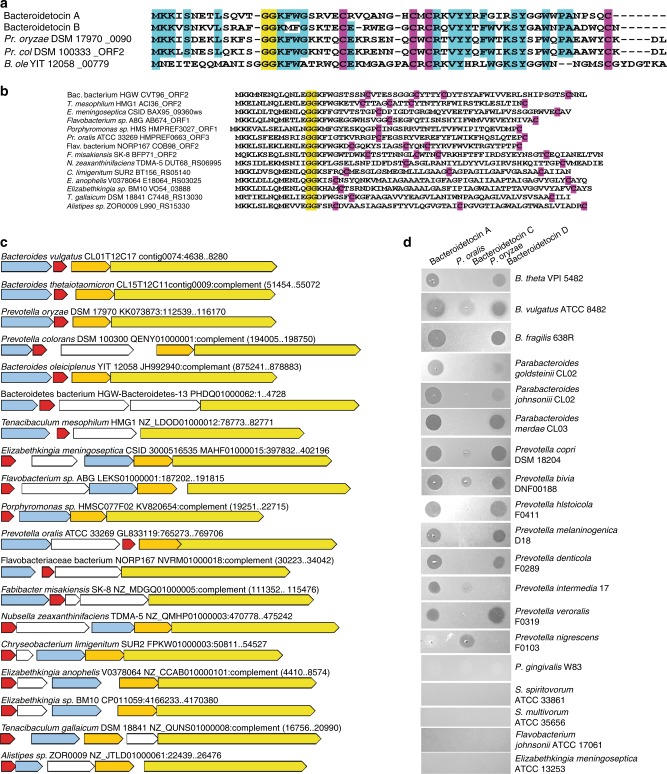
Additional peptides encoded by Bacteroidetes genomes with bacteroidetocin-like properties. **a** Alignment of peptides that were retrieved by tblastn searches of the Bacteroidetes/Chlorobi superphylum genomes using bacteroidetocin A and B (including leader sequences) as queries. The predicted GG cleavage site is highlighted yellow. Cysteine residues are highlighted pink, and residues conserved in at least four of the five peptides are highlighted in turquoise. *Pr*- *Prevotella*, ole – *oleiciplenus*, col – colorans. **b** Peptide sequences of potential bacteroidetocins retrieved using tblastn repeatedly in an iterative manner. Double GG cleavage motif is highlighted yellow and cysteine residues are highlighted pink. **c** ORF maps of the 19 genetic regions of each of the non-redundant bacteroidetocin-like peptides. The bacteroidetocin-like genes are colored red, TM protein genes are colored light blue, thiol oxidoreductase genes are colored gold, and ABC transporter/peptidase genes are colored yellow. Genes colored white are distinct from the four genes of the bacteroidetocin A and B regions. **d** Toxin dot assays of synthesized peptides bacteroidetocin A (left), the *Prevotella oralis* ATCC 33269 HMPREF0663_ORF3 peptide (bacteroidetocin C) (middle), and the *Prevotella. oryzae* DSM 17970 XylorDRAFT_0090 peptide (bacteroidetocin D) (right). 1 µg of peptide was applied in a 5 µl spot on top of an agar overlay containing the indicated strains

To determine if there are other bacteroidetocin-like peptides encoded by sequenced genomes from the Bacteroidetes/Chlorobi superphylum, we first used the full-length sequences of bacteroidetocin A and B as tblastn queries to detect small peptides that may not have been annotated. This analysis revealed three new peptides (Fig. 8a). Each of these peptides has a leader sequence of 15–16 aa including the putative GG cleavage site and the mature peptides are between 42 and 48 aa. In addition, each of these peptides contains four cysteine residues with similar positioning. These three new peptides were then further used in tblastn searches and those proteins retrieved were further used as queries until no further proteins were retrieved. Only those peptides with characteristics of bacteroidetocin A and B (small peptides with a GG motif near N-terminus) were retained, resulting in the retrieval of bacteroidetocin-like peptides from 35 genomes of four different classes of Bacteroidetes (Supplementary Data 2). The list of peptides was made non-redundant based on those clustering with 100% protein identity, reducing the list to 14 additional putative bacteroidetocins (Fig. 8b), for a total of 19 unique peptides. The genetic regions surrounding the new putative bacteroidetocins were analyzed and found to contain not only a putative bacteroidetocin-encoding gene, but additional genes encoding orthologs of the TM protein, the ABC transporter/peptidase, and, in most regions, the thiol oxiidoreductase (Fig. 8c). An amino-acid level similarity matrix of the four proteins encoded by these regions in comparison to the similar protein of each of the other regions is shown in Supplementary Data 2. The full-length similarity of many of these peptides with bacteroidetocins A and B is somewhat weak; however, all have similar leader sequences of 15–16 aa and at least two cysteine residues in addition to having the expected ancillary genes in close proximity.

We predicted that some of these newly identified peptides may also have toxin activity, especially those most similar to bacteroidetocin A and B. We chose two of these peptides for analysis based on their predicted solubility in water and based on the originating strains being isolated from diverse ecosystems. The peptides encoded by the genomes *Prevotella oryzae* DSM 17970, isolated from a flooded anoxic rice field in Japan, and *Prevotella oralis*, isolated from a human with periodontal disease, were chosen. Both of these peptides were chemically synthesized and tested for growth inhibition against a panel of diverse Bacteroidetes species and compared to the zones generated by the equivalent concentration of bacteroidetocin A (Fig. 8d). The *P. oralis* peptide, which unlike bacteroidetocin A and B has an uneven number of cysteines, showed only modest activity against a few of the strains tested; however, it is the only peptide of the three to have activity against *P. nigrescens*. This peptide was named bacteroidetocin C. The *P. oryza* peptide is broadly inhibitory, however, its pattern of inhibition is sporadic within a genus, targeting some *Bacteroides*, *Parabacteroides* and *Prevotella* species, but not others. This peptide was named bacteroidetocin D. We also tested the activity of these peptides against numerous Gram-positive and Gram-negative bacterial pathogens of other phyla (Supplementary Table 1), including *Listeria monocytogenes*, which is targeted by nearly all class IIa bacteriocins, and found no growth inhibition in the agar overlay assay (Supplementary Fig. 3), suggesting that the bacteroidetocins are likely specific for killing members of the Bacteroidetes phylum.

## Discussion

The bacteroidetocins identified in this study are very different from the other previously identified diffusible antibacterial toxins produced by the *Bacteroides*. The bacteroidetocins are broad-spectrum toxins that kill not only across species and genera, but also across families. The bacteroidetocins have several properties in common with class IIa bacteriocins, including similar size (35–50aa), the presence of at least two cysteines, and a leader sequence that is cleaved following a double glycine motif. However, there are numerous differences between the class IIa bacteriocins and the bacteroidetocins. Class IIa bacteriocins are produced by and target Gram-positive bacteria whereas our analysis of the bacteroidetocins suggest specificity for Bacteroidetes. Class IIa bacteriocins and bacteroidetocins have very little sequence similarity. The leader sequences of the bacteroidetocins are 15–16 aa whereas the leader sequences of the class IIa bacteriocins are typically 18–24 aa^28^. In addition, most of the residues of the conserved class IIa bacteriocin N-terminal motif YGNGVXC^20^ are missing in the bacteroidetocins, and alignments suggest that bacteroidetocins have distinct conserved motifs. Also, class IIa bacteriocins target the mannose phosphotransferase system in the membrane of Gram-positive bacteria^29–32^ leading to membrane permeabilization, collapse of the proton motive force, and loss of intracellular ATP^33,34^. *Bacteroides* species do not have a mannose phosphotransferase system, rather hexoses are transported across the inner membrane and phosphorylated by separate cytoplasmic kinases^35^. Therefore, the receptor(s) and the mechanism of action of the bacteroidetocins is still unknown. There are certainly also differences in the secretion and transport of these two types of molecules as the bacteroidetocins may have to transverse the Gram-negative outer membrane. In addition, there are several differences in the biosynthesis regions of these two types of bacteriocins that further differentiate these molecules. Based on these differences, the bacteroidetocins are a new type of bacteriocin and we propose they be designated class IIf.

The presence of four cysteine residues in bacteroidetocin A and B, their altered migration under non-reduced and reduced conditions and the presence of a thiol oxidoreductase encoding gene in these regions strongly suggests that they are disulfide bonded. The chemically synthesized peptide toxins used in our assays were synthesized without procedures for the formation of disulfide bonds and they have potent activity. An analysis of pediocin showed that linear analogs lacking disulfide bonds were as potent as the native disulfide bridged toxin^25^. Similar analyses of the bacteroidetocins may reveal that the disulfide bonds facilitate secretion of the protein, may help stabilize the protein, or protect it from degradation rather than directly affecting activity; however, this is yet to be determined.

The dynamics and spatial distribution of the toxins following release from the producing cell in the gut are certainly interesting topics for future study. It is possible that only a subset of cells are producing the bacteroidetocins at any given time, similar to some colicin-producing *E. coli* strains^36,37^. The production of bacteroidetocins by only a subpopulation may contribute to the persistence of bacteroidetocin-producing strains in the human gut and other environments. In addition, our data do not rule out the possibility that the cells may not actively secrete the toxin across the outer membrane and instead, the bacteria may need to lyse to release the toxin. Cell lysis is required for the release of many colicins^38–41^.

Our query for additional bacteroidetocin-like peptides revealed 17 other peptides encoded by Bacteroidetes genomes (Fig. 8a, b). These peptides are all of similar size, have similar leader peptides, have at least two cysteine residues, and all are encoded by regions that have a gene encoding a similar ABC transporter/peptidase and TM protein and most also include a thiol oxidoreductase gene (Fig. 8c). We showed that the peptide from *P. oryzae* (bacteroidetocin D) has broad-spectrum anti-Bacteroidales activity whereas the peptide from *P. oralis* (bacteriodetocin C) has more specific killing affecting only a few of the tested strains. The mechanism resulting in differential targeting by these various bacteroidetocins is currently unknown and may be due to a specific outer membrane protein/transporter or an inner membrane or cytoplasmic target. It is likely that the collective group of putative bacteroidetocins identified in this study, as well as others revealed as more Bacteroidetes strains are sequenced, will allow for targeting of a broader range of Bacteroidetes strains by this class of toxin.

The bacteroidetocins offer numerous potential translational applications. Bacteriocins have long been studied as tools for food preservation and in the fight against human pathogens, especially as antibiotic resistance increases^42,43^. The bacteroidetocins target a diverse phylum of bacteria not previously shown to be targeted by other peptide bacteriocins. These include important bacterial pathogens involved in polymicrobial infections of the female reproductive tract and of the oral cavity. As the cellular features that determine strain sensitivity and the peptide residues that dictate specificity are identified, we should be able to use these peptides or modified versions of them to specifically target particular organisms for microbiome engineering and therapeutic purposes.

## Methods

### Bacterial strains and growth media

All strains used in this study are listed in Supplementary Table 1 as are the media used for their growth. Basal medium contains 2 g proteose peptone, 0.5 g yeast extract, 0.5 g NaCl per 100 ml with the addition of glucose to 0.5%, K_2_HPO_4_ to 0.5%, cysteine to 0.05%, hemin 5 µg/ml, and 0.25 µl/100 ml Vitamin K_1_. Amended TSA or TSB (TSA-A or TSB-A) contains per liter 30 g trypticase soy (agar or broth), 5 g yeast extract, 0.5 g cysteine-HCl, with the addition of hemin and vitamin K_1_ following autoclaving. All Bacteroidetes strains were grown at 37 °C under anaerobic conditions except for *S. multivorum*, *S. spiritovorum*, *E. meningogenica*, and *F. johnsoniae*, which were incubated at 30 °C in air. Antibiotics used for cloning and transposon mutagenesis were at the following concentrations: carbenicillin 100 µg/ml, kanamycin 50 µg/ml, gentamicin 200 µg/ml, erythromycin 5 µg/ml, tetracycline 5 µg/ml.

### Transposon mutagenesis

Transposon mutagenesis was performed using strain *B. ovatus* CL02T12C04 and the transposon vector pYT646b^44^. Tetracycline-resistant transposon mutants were screened for loss of toxin activity in agar spot overlay assays using *B. thetaiotaomicron* VPI-5482 as the overlay strain. The junctions of mutants lacking secreted toxin activity were cloned using the resulting *E. coli* origin of replication and β-lactamase resistance gene from the transposon vector^44^. The junctions were identified using a primer reading out of the transposon into the chromosomal DNA (Supplementary Table 2).

### Deletion mutation and complementation

Internal deletion mutants of EH213_01843 (thiol oxidoreductase), EH213_01844 (bacteroidetocin B) and EH213_01845 (TM protein) were constructed in strain BtCL15T12C11. DNA segments upstream and downstream from the regions to be deleted were PCR amplified with Phusion polymerase (New England Biolabs NEB) using the primers listed in Supplementary Table 2, digested with BamHI and MluI (1844, 1845) and cloned into pNJR6^45^. For the EH213_01843, DNA was amplified and cloned into the PstI site of pLGB13^46^ using NEBuillder (NEB). Plasmids were transferred into BtCL15T12C11 by triparental mating with helper plasmid RK231 or mating from *E. coli* S17 ʎpir, and cointegrates were selected on gentamicin/erythromycin plates. Following growth under nonselective conditions, erythromycin-sensitive colonies were screened by PCR for the mutant genotype.

EH213_01843, 1844, and 1845 were PCR amplified using Phusion polymerase (NEB) with primers listed in Supplementary Table 2. Products were digested with BamHI, gel purified, and cloned into the BamHI site of *Bacteroides* expression vector pFD340^47^ and screened for proper orientation of the genes in relation to the vector-borne promoter. Plasmids were mated into *Bacteroides* strains using conjugal helper plasmid RK231. The inserts of all plasmids were sequenced to confirm no mutations were incorporated.

### Agar overlay assays – toxin dot assays

The agar spot overlay assay^48^ was modified using two successive applications of Whatman filter paper to the plates to remove the dotted strains (those testing for toxin production) prior to exposure to chloroform vapor for 15 min and then application of the second strain in top agar. The second strain was applied in 4 ml of top agar for standard 9 cm diameter petri dishes and in 6 ml of top agar for the 8 cm × 12 cm rectangular plates. For analysis of synthesized peptides, 5 µl of the peptide at indicated concentrations were added on top of the overlay and incubated anaerobically overnight. All peptides were synthesized by LifeTein (Hillsborough, NJ), solubilized in distilled water and filter sterilized. The water only control did not affect growth of any bacteria.

### Expression of genes in *E. coli*

The four-gene region encompassing EH213_01845-42 of strain BtCL15T12C11 was PCR amplified using Phusion polymerase with the primers listed in Supplementary Table 2. This DNA was gel purified and cloned into the NcoI site of pET16b (Novagen) using NEBuilder (NEB) according to manufacturer’s specifications and transformed into *E. coli* BL21/DE3. Transformants were PCR-screened to confirm the presence of the insert and the resulting plasmid was subjected to whole plasmid sequencing at the Massachusetts General Hospital CCIB DNA Core facility. *E. coli* BL21/DE3 containing pET16b or pET16b with the cloned genes was spotted onto plates with or without added IPTG and grown overnight aerobically. The *E. coli* were removed, the plate was exposed to chloroform vapor, overlaid with *B. thetaiotaomicron* VPI-5482, and incubated overnight anaerobically.

### Effects of bacteroidetocin A on bacterial growth in broth

Bacteria were swabbed from a fresh plate into pre-reduced media at an OD_600_ of approximately 0.6–0.8. In total, 10 µl of this bacterial suspension was added to 100 µl of media containing either 10 µl of added water or 10 µl of 3853 peptide (2 µg total peptide added). Bacteria were incubated and OD_600_ readings were recorded over time under anaerobic conditions using an Eon high-performance microplate spectrophotometer (BioTek Instruments, Winooski, VT). Data are shown as the mean of biological triplicates with the SEM plotted as error bars, as calculated by Prism version 8.0.1 for 64-bit Windows (GraphPad Software, San Diego, CA).

For calculations of bacteroidetocin effect on bacterial viability, the experiments were performed as above except the starting cultures (T = 0) were plated for enumeration and wells containing bacteria exposed to toxin (biological triplicates) were plated after four hours and compared to the cfu of bacteria in a well treated only with the water control.

### Analysis of bacteroidetocin A and B from wild-type bacteria

For analysis of native bacteroidetocin A and B, BvCL01T12C17, BtCL15T12C11, and BtCL15T12C11∆1844 were grown anaerobically as a lawn on BHIS agar plates for two days. Bacteria and associated toxin were swabbed from the plates and resuspended in PBS. The bacteria were vortexed for one minute to remove cell associated toxin and the bacteria were pelleted by centrifugation and the supernatants were collected. Some of the sample was treated with 5% β-mercaptoethanol (BME) or an equivalent amount of PBS for the non-reduced samples and incubated at room temperature for 30 min. NUPAGE LDS sample buffer (Invitrogen) was added to the samples and they were boiled for 5 min, then run on a 12% Bolt Bis/Tris plus gel (Invitrogen). For western immunoblot analysis, polyclonal antiserum to the mature 42 amino acid bacteroidetocin A and B peptides were raised in rabbits by Lampire Biological Laboratories (Pipersville, PA) using the ExpressLine protocol. Contents of the gels were transferred to PVDF membrane and probed with the antisera at a dilution of 1:20. Alkaline phosphatase labeled goat-anti rabbit antibodies (Thermo Scientific, Waltham, MA) were used as the secondary antibody and the blots were developed with BCIP/NBT (KPL, Gaithersburg, MD). Uncropped, unprocessed blots are contained in the Source Data file.

### LC–MS/MS analysis of bacteroidetocin A and B

Liquid chromatography with tandem mass spectrometry (LC–MS/MS) analysis of in vivo produced bacteroidetocin A and B peptides was performed by the Taplin Biological Mass Spectrometry Facility at Harvard Medical School, Boston, MA. Bacteroidetocin A and B were prepared as described above except that the supernatants following vortex and removal of the cells were concentrated 20-fold using an Amicon Ultra 3 K centrifugal filter. Some of the concentrated samples were reduced with 5% BME as described above. All samples were treated with iodoacetic acid (IAA) at a final concentration of 10 mM for one hour at room temperature to alkylate free cysteines. LDS sample buffer was added, samples were boiled for 5 min and run on a 12% Bolt Bis/Tris plus gel (NUPAGE). Gels were washed with deionized water several times and stained with Imperial Protein Stain (Thermo Fisher). Gels were destained and bands were excised and cut into approximately 1 mm^3^ pieces, subjected to a modified in-gel trypsin digestion procedure^49^, washed and dehydrated with acetonitrile for 10 min and completely dried in a speed-vac. The gel slices were rehydrated in 50 mM ammonium bicarbonate solution containing 12.5 ng/µl modified sequencing-grade trypsin (Promega, Madison, WI) at 4 °C for 45 min. The trypsin solution was replaced with enough 50 mM ammonium bicarbonate solution to just cover the gel pieces, and the samples were held at 37 °C overnight. Peptides were extracted from the gel slices, washed once in 50% acetonitrile and 1% formic acid, and dried in a speed-vac for ~1 h. The dried peptide samples were reconstituted in 5–10 µl of 2.5% acetonitrile, 0.1% formic acid. A nano-scale reverse-phase HPLC capillary column was created by packing 2.6 µm C_18_ spherical silica beads into a fused silica capillary (100 µm inner diameter × ~30 cm length) with a flame-drawn tip^50^. Each sample was loaded on the equilibrated column via a Famos auto sampler (LC Packings, San Francisco CA). A gradient was formed and peptides were eluted with increasing concentrations of 97.5% acetonitrile, 0.1% formic acid, subjected to electrospray ionization, and entered into an LTQ Orbitrap Velos Pro ion-trap mass spectrometer (Thermo Fisher Scientific, Waltham, MA). Peptides were detected, isolated, and fragmented to produce a tandem mass spectrum of specific fragment ions for each peptide. Peptide sequences (and hence protein identity) were determined by matching the acquired fragmentation pattern with protein databases using the software program Sequest (Thermo Fisher Scientific, Waltham, MA)^51^. All databases included a reversed version of all the sequences and the data was filtered to between a one and two percent peptide false discovery rate.

### Mouse colonization studies and western blot analysis

Mouse studies were approved by the Institutional Animal Care and Use Committee (IACUC), Brigham & Women’s Hospital and comply with all relevant ethical regulations for animal testing and research. Six female and six male Swiss Webster germ free mice 8 weeks old were obtained from and maintained in the Harvard Digestive Diseases Center gnotobiotic core facility at Brigham and Women’s Hospital, Boston, MA. Groups of three sex-matched mice were housed in sterile OptiMICE cages (Animal Care Systems, Centennial, CO). Mice were gavaged with 200 µl of bacteria swabbed from a plate (to a final OD_600_ of approximately 0.4) into medium to ensure viability of the toxin-producing strain. Bacteria were either wild-type BtCL15T12C11 or the isogenic deletion mutant lacking the EH213_01844 bacteroidetocin B gene. After six days, fecal samples were collected and concentration of viable bacteria was quantified by dilution plating. A two-tailed Mann-Whitney test as calculated by Prism version 8.0.1 for 64-bit Windows (GraphPad Software, San Diego, CA) was used for statistical analysis of colonization levels between the two groups.

Mouse fecal samples from the above experiment were diluted 1:10 in PBS and allowed to separate by gravity for 5 min to separate fibrous material, then, 20 µl from the top material was run on a 12% SDS-PAGE Bis/Tris gel (Invitrogen) and immunoblotted and probed with antibodies as described above. As the CFU/g of bacteria in each sample was the same, sample loading was controlled by loading the same volume of sample per well.

### Genomes included in in silico analyses

Our locally curated Bacteroidetes/Chlorobi group genome set includes all genomes identified by NCBI as belonging to the “Bacteroidetes/Chlorobi group” as of October 8, 2018, that contained a depositor-supplied protein translation file, with the addition of nine *Bacteroides* genomes sequenced in-house. The collection utilized thus comprises 3,663 genomes, encoding 11,741,790 annotated genes

### Identification of bacteroidetocin biosynthesis regions

As the open reading frames encoding bacteroidetocin A and B may not have been annotated as coding domains in genomes due to their small size, we used tblastn^52^ in an iterative manner, using the peptide sequences of EH214_03825 (bacteroidetocin A; from *B. vulgatus* CL01T12C17) and EH213_01844 (bacteroidetocin B; from *B. thetaiotaomicron* CL15T12C11) as initial queries. Each significant return containing an ORF encoding a bacteroidetocin-like product was then translated, and this amino acid sequence was used as an additional tblastn query until convergence was achieved.

The DNA coordinates indicated by the tblastn returns were expanded to include 10,000 bp on either side of the hit, or as much as was available on any particular scaffold. If the region contained an ORF potentially encoding a bacteroidetocin-like peptide, genes in close proximity were further analyzed and those that contained one or more of the expected ancillary export machinery proteins (a protein with TM regions across its entire length, a thiol oxidoreductase, and/or a peptidase domain-containing ABC transporter) were retained for further study. Thirty-five such regions were detected, encoding thirty-six potential bacteroidetocins. ORFs detected as encoding potential bacteroidetocins were not annotated as coding domain sequences in 45.7% of the identified regions (16 of 35). This collection of potential bacteroidetocin peptides was made non-redundant at 100% amino acid identity, resulting in 17 newly identified unique peptide sequences (Fig. 8a, b, Supplementary Data 2).

### Genome sequencing

Chromosomal DNA from *B. vulgatus* CL01T12C17, *B. vulgatus* CL14T03C19, and *B. thetaiotaomicron* CL15T12C11 was recovered using the Qiagen G-100 kit, fragmented using a Covaris S2 instrument, and analyzed for appropriate fragment distribution with a high-sensitivity D1K TapeStation machine and for sufficient quantity by a SYBR quantitative PCR (qPCR) assay. Paired-end reads of 150 bp were generated by the Biopolymers Facility, Harvard Medical School, using an Illumina MiSeq sequencer. BBDuk (version 37.95, part of the BBTools suite of programs distributed by the Department of Energy’s Joint Genome Institute; https://jgi.doe.gov/data-and-tools/bbtools/) was used to remove adapter sequences and quality trim the raw Illumina reads. The resulting reads were further screened via blastn against NCBI’s UniVec_Core collection (build 10.0, with entries originating in GenBank removed), and reads returning a significant hit were discarded before assembly. Velvet Optimizer (version 2.2.5, http://www.vicbioinformatics.com/software.velvetoptimiser.shtml) was utilized to determine the optimal settings (e.g. k value) for assembly, and de novo assembly was performed using Velvet 1.2.11^53^. The draft genomes were annotated using an in-house-customized version of Prokka (version 1.12)^54^. The three genomes described herein were deposited in GenBank with accession numbers provided in the ‘Data availability’ section.

### Human gut metagenomic analyses

The “3 consortium gene catalog” (3GCG) subset of the integrated gene catalog (IGC)^27^ comprises 1,267 samples of human gut metagenomes from 1,070 unique individuals. The protein sequences of EH214_03825 (bacteroidetocin A, from *B. vulgatus* CL01T12C17) and EH213_01844 (bacteroidetocin B, from *B. thetaiotaomicron* CL15T12C11) were used as tblastn queries against a DNA BLAST database comprised of all 62,204,620 contigs and scaffolds (132,484,518,450 bases) included in this metagenomics data set. The amino acid sequences of the two queries are similar enough to one another that each returned a hit to the same contig and region, albeit at a different percent similarity. The lower quality duplicate high-scoring local segment (generally ~52% similarity) was discarded. The retained hits were then further reduced by identifying repetitive samples from the same individual, and retaining only one. Comparisons of the distribution of bacteroidetocin A or B detected between unique metagenomic samples from nationalities and groups of nationalities (Table 1, Supplementary Data 1) were performed using a two-sided Fisher’s exact test as implemented in Prism version 8.0.1 for 64-bit Windows (GraphPad Software, San Diego, CA), using an alpha level of 0.01.

### Sequence alignments, ORF maps, and similarity matrices

The similarity matrices included as Supplementary Data 2 were generated for full-length proteins by performing a global comparison of each member of a protein class (TM proteins, bacteroidetocins, thiol oxidoreductases, and ABC-type bacteroidetocin transporters) with all other members of the same class using the Needleman-Wunsch algorithm^55^ as implemented by needle (part of the EMBOSS package, version 6.6.0^56^) using the EBLOSUM62 substitution matrix and with the endweight switch enabled. Comparisons involving protein sequences translated from truncated genes (DWZ35_246000p, DXA67_22310p, CVT96_05470p, and COB98_ORF1p, all encoding TM proteins), are reported as similarity percentages based on local alignments performed using the Smith-Waterman algorithm^57^ as implemented by the EMBOSS program water, using the same EBLOSUM62 substitution matrix.

Examination of several loci revealed likely errors (frameshifts, likely incorrectly called start codons, and missing open reading frames). The frameshifts were all single base indels in homopolymeric regions, and often resulted in two adjacent and overlapping smaller ORFs that each had protein-level similarity with the same expected (longer) target. Suspected incorrect start codons were also detected and corrected. Open reading frames not detected by the gene-calling software employed during annotation of the genome were also encountered, most frequently with regard to the small bacteroidetocin-encoding ORFs. We speculatively corrected these putative defects before generating the similarity matrices (Supplementary Data 2) and open reading frame maps (Fig. 8c).

In the case of a frameshift correction, the gene name used here consists of the base name of the furthest upstream gene involved with the suffix ‘fs’ appended. Similarly, the suffixes ‘ws’ (wrong start) and ‘p’ (partial, due to the reading frame beginning before or ending after a contig or scaffold termini) were used. A previously uncalled open reading frame was annotated using the genome’s locus tag prefix, followed by ‘ORF’ and a digit indicating its position in the gene order of the locus map used here.

### Other software used

Multiple sequence alignments were routinely generated using Clustal Omega^58^ or MAFFT^59^ and colored alignments were generated with a slightly modified (for the color table) version of MView^60^; all such software was used either as command-line utilities or as implemented by the website of the European Molecular Biology Laboratory’s European Bioinformatics Institute (EMBL-EBI)^61^. Transmembrane regions were determined using TMHMM 2.0c^62^

### Ethical compliance

All applicable research described in this paper, including recombinant DNA work and animal studies, was performed in adherence to all ethical regulations, and was reviewed and approved by the Partners Institutional Biosafety Committee (PIBC), Partners HealthCare System, Boston, MA, and/or the Institutional Animal Care and Use Committee (IACUC), Brigham & Women’s Hospital, Boston, MA.

### Reporting summary

Further information on research design is available in the Nature Research Reporting Summary linked to this article.

## Supplementary information

Supplementary Information

Description of Additional Supplementary Files

Supplementary Data 1

Supplementary Data 2

Reporting Summary

Source Data

## Data Availability

Data underlying Figs 3c, 3d, 4b, 4c, 4e, 5b, 6a, 6b, and Supplementary Fig. 2 are provided as Source Data files. The metagenomics data sets containing all the raw data and associated metadata underlying Supplementary Data 1 are available as detailed in ref. ^27^. The three genomes described herein were deposited in GenBank under the following identifying numbers: *Bacteroides thetaiotaomicron* CL15T12C11, Genbank accession RWHY00000000, BioProject ID PRJNA506939, BioSample ID SAMN10476872; *Bacteroides vulgatus* CL01T12C17, Genbank accession RWHZ00000000, BioProject ID PRJNA506941, BioSample ID SAMN10476933; and *Bacteroides vulgatus* CL14T03C19 Genbank accession RWIA00000000, BioProject ID PRJNA506943, BioSample ID SAMN10477097. The authors declare that all other data supporting the findings of this study are available either within the paper (and its Supplementary Information files), or from the corresponding author on request.
