# Supplementary material for: A family of anti-Bacteroidales peptide toxins wide-spread in the human gut microbiota
Source: Nat Commun. 2019 Aug 1;10:3460. doi: 10.1038/s41467-019-11494-1 (PMC6671954; doi:10.1038/s41467-019-11494-1)
Supplement: Supplementary file 1 — Supplementary Information [file 41467_2019_11494_MOESM1_ESM.pdf]

Supplementary Information

A family of anti-Bacteroidales peptide toxins wide-spread in the human gut microbiota

Coyne, Béchon, *et al.*

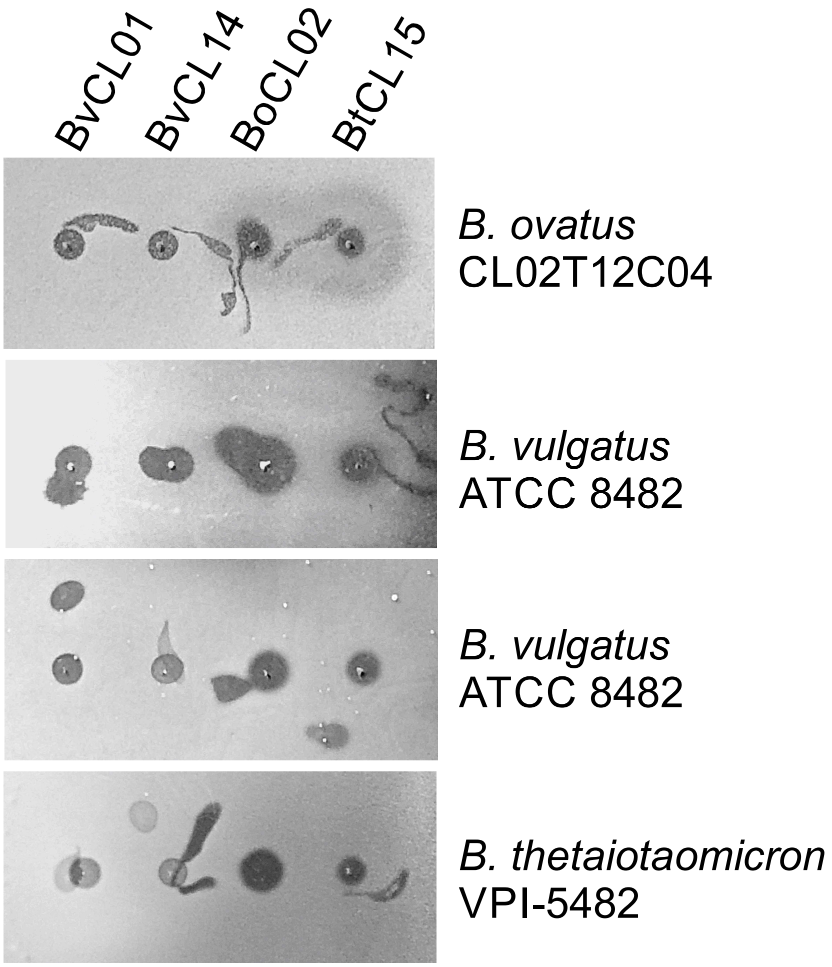

Supplementary Figure 1. Agar spot overlay assays demonstrating the movement of the toxin on the agar surface due to physical application of the overlay. The four toxin-producing strains are shown at the top and were cultured on the plate overnight, removed, then the plate was overlaid with top agar containing the strains shown on the left. Representative overlays are shown to illustrate the effect.

EH214_03825 *B. vulgatus* CL01T12C17 (bacteroidetocin A)

M K K I S N E T L S Q V T G G K F W G S R V E C R V Q A N G H C M C R K V Y Y R F G I R S Y G G W W P A N P S Q C	no. times seen
K F W G S	2
K F W G S R	55
K F W G S R V E	1
F W G S R	12
F W G S R V E	1
V Q A N G H	11
K V Y Y R	170
V Y Y R	89
V Y Y R F	1
V Y Y R F G I R S Y G G W	1
Y R F G I R	1
R F G I R	2
F G I R S Y G G W W	1
S Y G G W W P A	1
S Y G G W W P A N	3
S Y G G W W P A N P	2
S Y G G W W P A N P S	2
S Y G G W W P A N P S Q	57
S Y G G W W P A N P S Q C	9
Y G G W W P A N P S	1
Y G G W W P A N P S Q	1
G G W W P A N P S Q	1
G W W P A N P S Q	1

EH213_01844 *B. thetaiotomicron* CL15T12C11 (bacteroidetocin B)

M K K V S N K V L S R A F G G K M F G S K T E C E R W E G G C R C R Q V Y Y T F W V K S Y G A W N P A A D W Q C N	no. times seen
K M F G S K	11
K M F G S K T E C E R	9
Q V Y Y T F	1
Q V Y Y T F W V K	21
Y T F W V K	2
T F W V K	1
S Y G A W N P A	1
S Y G A W N P A A D	2
S Y G A W N P A A D W Q	4
S Y G A W N P A A D W Q C N	6
G A W N P A A D W Q C N	1

Supplementary Figure 2. Fragments of bacteroidetocin A and B identified by LC-MS/MS analysis. Yellow highlight indicate residues not detected. Blue highlights are completely or partially digested tryptic fragments. Numbers on the right indicate the number of times the fragment was detected.

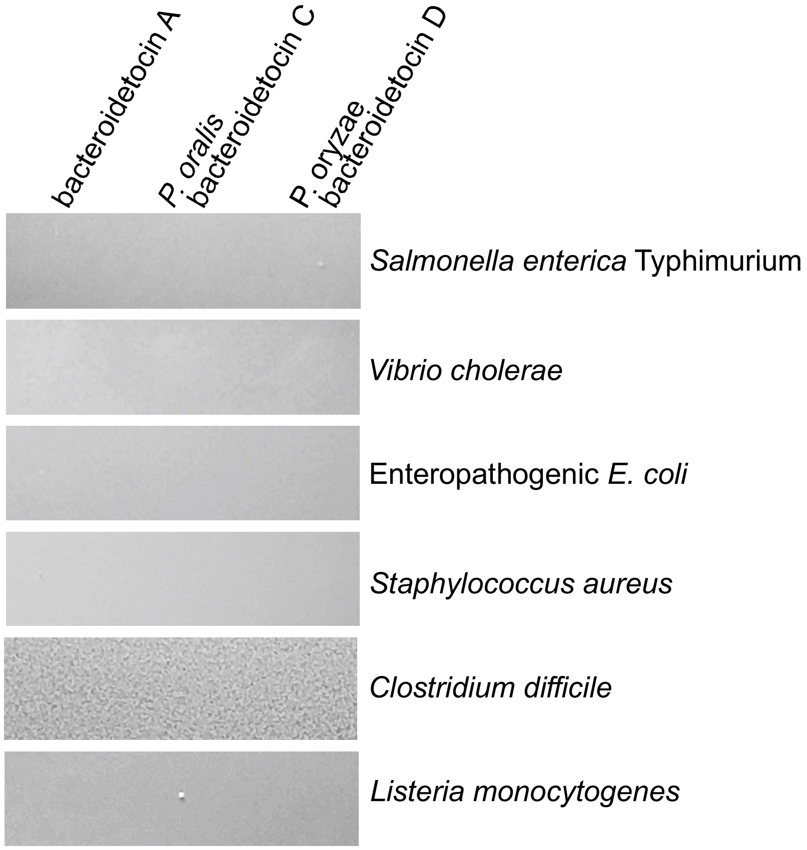

Supplementary Figure 3. Toxin dot assays of synthesized peptides bacteroidetocin A (left), the *Prevotella oralis* ATCC 33269 HMPREF0663_ORF3 peptide (bacteroidetocin C) (middle), and the *Prevotella oryzae* DSM 17970 XylorDRAFT_0090 peptide (bacteroidetocin D) (right) against bacterial pathogens of non-Bacteroidetes phyla. 1 µg of peptide was applied in a 5 µl spot on top of an agar overlay containing the indicated strains.

Supplementary Table 1. Strains used in this study.

**Genome name**	**media liquid/solid**	**source**
Bacteroides vulgatus CL01T12C17	basal/BHIS	Comstock Lab
Bacteroides vulgatus CL14T03C19	basal/BHIS	Comstock Lab
Bacteroides ovatus CL02T12C04	basal/BHIS	Comstock Lab
Bacteroides ovatus CL02T12C04 tn mut 206	basal/BHIS	Comstock Lab
Bacteroides ovatus CL02T12C04 tn mut 512	basal/BHIS	Comstock Lab
Bacteroides ovatus CL02T12C04 tn mut 363	basal/BHIS	Comstock Lab
Bacteroides ovatus CL02T12C04 tn mut 724	basal/BHIS	Comstock Lab
Bacteroides thetaiotaomicron CL15T12C11	basal/BHIS	Comstock Lab
Bacteroides thetaiotaomicron CL15T12C11 Δ1843	basal/BHIS	Comstock Lab
Bacteroides thetaiotaomicron CL15T12C11 Δ1844	basal/BHIS	Comstock Lab
Bacteroides thetaiotaomicron CL15T12C11 Δ1845	basal/BHIS	Comstock Lab
Bacteroides thetaiotaomicron VPI 5482	basal/BHIS	ATCC
Bacteroides vulgatus ATCC 8482	basal/BHIS	ATCC
Bacteroides fragilis 638R	basal/BHIS	lab collection
Parabacteroides merdae CL03T12C32	basal/BHIS	Comstock Lab
Parabacteroides johnsonii CL02T12C29	basal/BHIS	Comstock Lab
Parabacteroides goldsteinii CL02T12C30	basal/BHIS	Comstock Lab
Prevotella melaninogenica D18	TSB-A/TSA-A	BEI
Prevotella denticola F0289, Oral Taxon 291	TSB-A/TSA-A	BEI
Prevotella nigrescens F0103	TSB-A/TSA-A	BEI
Prevotella histicola F0411	TSB-A/TSA-A	BEI
Prevotella oralis HGA0225	TSB-A/TSA-A	BEI
Prevotella bivia DNF00188	TSB-A/TSA-A	BEI
Prevotella veroralis F0319	TSB-A/TSA-A	BEI
Prevotella intermedia str. 17	TSB-A/TSA-A	lab collection
Prevotella copr i DSM 18205	TSB-A/TSA-A	DSM
Porphyromonas gingivalis W83	TSB-A/TSA-A	lab collection
Sphingobacterium spiritovorum ATCC 33861	BHIS	ATCC
Sphingobacterium multivorum ATCC 35656	BHIS	ATCC
Flavobacterium johnsoniae ATCC 17061	BHIS	ATCC
Elizabethkingia meningoseptica ATCC 13253	BHIS	ATCC
Escherichia coli HS	BHIS	lab collection
Lactobacillus rhamnosus LMS2-1	BHIS	BEI
Bifidobacterium breve EX336960VC18	BHIS	BEI
Enterococcus faecium 503	basal/BHIS	BEI
Salmonella enterica Typhimurium ATCC 14028	BHIS	ATCC
Enteropathogenic *E. coli* 2348/69	BHIS	lab collection
Vibrio cholerae N16961	BHIS	lab collection
Staphylococcus aureus 502A	TSA-A	J. Lee
Clostridium difficile NAP07	BHIS	BEI
Listeria monocytogenes 104035	BHIS	M. Waldor
Escherichia coli DH5α	LB	lab collection
Escherichia coli BL21/DE3	LB	lab collection
Escherichia coli S17 λ pir	LB	E. Martens

Supplementary Table 2. Primers used in this study.

Purpose	Primer Sequence [a]	Orientation
Sequence out of pYT646 junction	GACTTGGATACCTCACGCC	
Cloning of EH213_01845	AACGGGATCCATAGCAAAACTTCATGCGTAGCAA<br>TTCAGGATCCCGGGACAAAACTTTGTTGCTTACTT	Forward<br>Reverse
Cloning of EH213_01844	TTTTGGATCCGGAATAATAACAACCTTTATTGGTTTT<br>GTCAGGATCCATTTACCTACTGGTTAGTTGCACTG	Forward<br>Reverse
Cloning of EH213_01843	TTATGGATCC GTAAAGCCAATTGATGGTATATCTCTTTTATT<br>TCGCGGATCCTGTTGCGTAAATACTTTCTTCGCT	Forward<br>Reverse
Deletion of EH213_01845	CATAGGATCCGATATTCGCCGTCTTTCATTCTAC<br>TGGTACGCGTTCAAATATTAAGTTTCCCAGTATGGT<br>TTTTACGCGTTCATACGGTATTGGTTTGTGTTTT<br>AGTTGGATCCTTTATGGATGCTTGAAGAATTTGA	left flank forward<br>left flank reverse<br>right flank forward<br>right flank reverse
Deletion of EH213_01844	AGAGGGATCCATACAGGGAAATACGGATTTGAGA<br>TTCAACGCGTGGGACAAAACTTTGTTGCTTACTT<br>AATAACGCGTCAGTGCAACTAACCAGTAGGTAAATAA<br>AATAGGATCCACCGATCCGACCAAATATAGATAA	left flank forward<br>left flank reverse<br>right flank forward<br>right flank reverse
Deletion of EH213_01843	AGTGGATCCCCCGGGCTGCACCATACTGGGAAACTTAATATTTG<br>TTAGTTGTTTTGTTGCAATACAATATTTTTTTCTATTC<br>TATTGCAACAAAACAACTAAAAAGTAATATGTAATGATATTAAATATC<br>CTTGATATCGAATTCCTGCAGACAACCAAATAAGCGAAG	left flank forward<br>left flank reverse<br>right flank forward<br>right flank reverse
Cloning 1845-1842 into into pET16b	ACTTTAAGAAGGAGATATAC<br>ATGCGTAGCAAGAAATATAAAAAATATATTTTG<br>CTGTGATGATGATGATGATGATGATGATGATGGCC<br>AATCGTCATAGCAACCAAC	Forward<br><br>Reverse

[a] Restriction sites used for cloning are underlined
