# Supplementary material for: A family of anti-Bacteroidales peptide toxins wide-spread in the human gut microbiota
Source: Nat Commun. 2019 Aug 1;10:3460. doi: 10.1038/s41467-019-11494-1 (PMC6671954; doi:10.1038/s41467-019-11494-1)
Supplement: Supplementary file 2 — Description of Additional Supplementary Files [file 41467_2019_11494_MOESM2_ESM.pdf]

Description of Additional Supplementary Files

File Name: Supplementary Data 1
Description: Human gut metagenomes of the 3CGC collection that contain the bacteriodetocin A (bacteroidetocin A tab) or the bacteriodetocin B (bacteroidetocin B tab) gene.

File Name: Supplementary Data 2
Description: Similarity matrices of each of the bacteroidetocin-related protein families (bacteroidetocins, non-redundant bacteroidetocins, TM proteins, thiol oxidoreductases, and ABC transporters) with each other.
