# Supplementary material for: A family of anti-Bacteroidales peptide toxins wide-spread in the human gut microbiota
Source: Nat Commun. 2019 Aug 1;10:3460. doi: 10.1038/s41467-019-11494-1 (PMC6671954; doi:10.1038/s41467-019-11494-1)
Supplement: Supplementary file 5 — Reporting Summary [file 41467_2019_11494_MOESM5_ESM.pdf]

nature research

Corresponding author(s): Laurie E. Comstock
lcomstock@rics.bwh.harvard.edu

Last updated by author(s): May 28, 2019

Reporting Summary

Nature Research wishes to improve the reproducibility of the work that we publish. This form provides structure for consistency and transparency in reporting. For further information on Nature Research policies, see Authors & Referees and the Editorial Policy Checklist.

Statistics

For all statistical analyses, confirm that the following items are present in the figure legend, table legend, main text, or Methods section.

n/a	Confirmed	
☐	☒	The exact sample size (*n*) for each experimental group/condition, given as a discrete number and unit of measurement
☐	☒	A statement on whether measurements were taken from distinct samples or whether the same sample was measured repeatedly
☐	☒	The statistical test(s) used AND whether they are one- or two-sided<br>*Only common tests should be described solely by name; describe more complex techniques in the Methods section.*
☒	☐	A description of all covariates tested
☒	☐	A description of any assumptions or corrections, such as tests of normality and adjustment for multiple comparisons
☐	☒	A full description of the statistical parameters including central tendency (e.g. means) or other basic estimates (e.g. regression coefficient) AND variation (e.g. standard deviation) or associated estimates of uncertainty (e.g. confidence intervals)
☐	☒	For null hypothesis testing, the test statistic (e.g. *F*, *t*, *r*) with confidence intervals, effect sizes, degrees of freedom and *P* value noted<br>*Give P values as exact values whenever suitable.*
☒	☐	For Bayesian analysis, information on the choice of priors and Markov chain Monte Carlo settings
☒	☐	For hierarchical and complex designs, identification of the appropriate level for tests and full reporting of outcomes
☒	☐	Estimates of effect sizes (e.g. Cohen's *d*, Pearson's *r*), indicating how they were calculated

Our web collection on statistics for biologists contains articles on many of the points above.

Software and code

Policy information about availability of computer code

Data collection	All commercial and open source software used is fully described in the Materials & Methods section of the manuscript.
Data analysis	The data analysis methodology used is fully described in the Materials & Methods section of the manuscript.

For manuscripts utilizing custom algorithms or software that are central to the research but not yet described in published literature, software must be made available to editors/reviewers. We strongly encourage code deposition in a community repository (e.g. GitHub). See the Nature Research guidelines for submitting code & software for further information.

Data

Policy information about availability of data

All manuscripts must include a data availability statement. This statement should provide the following information, where applicable:

- Accession codes, unique identifiers, or web links for publicly available datasets
- A list of figures that have associated raw data
- A description of any restrictions on data availability

The authors declare that the data supporting the findings of this study are available either within the paper (and its supplementary information files), or from the corresponding author on reasonable request.

Field-specific reporting

Please select the one below that is the best fit for your research. If you are not sure, read the appropriate sections before making your selection.

☒ Life sciences ☐ Behavioural & social sciences ☐ Ecological, evolutionary & environmental sciences

Life sciences study design

All studies must disclose on these points even when the disclosure is negative.

Sample size	In studies involving mice, the sample sizes were chosen to minimize the number of animals used while retaining the ability to calculate statistical significance. In bacterial growth rate experiments, the number of replicate cultures used (minimum of three) was selected based on experience.
Data exclusions	No data was excluded from this study.
Replication	Bacterial growth studies report the average +/- SEM of a minimum of three replicate cultures. Each strain (2) used in animal studies utilized three female and three male Swiss Webster germ free mice; results shown are the average +/- SEM.
Randomization	Randomization of samples in this study was not performed, as it would bring no additional strength to the conclusions.
Blinding	Blinding was not necessary in these studies, as no patient identifiable materials were used.

Reporting for specific materials, systems and methods

We require information from authors about some types of materials, experimental systems and methods used in many studies. Here, indicate whether each material, system or method listed is relevant to your study. If you are not sure if a list item applies to your research, read the appropriate section before selecting a response.

Materials & experimental systems

n/a	Involved in the study	
☐	☒	Antibodies
☒	☐	Eukaryotic cell lines
☒	☐	Palaeontology
☐	☒	Animals and other organisms
☒	☐	Human research participants
☒	☐	Clinical data

Methods

n/a	Involved in the study	
☒	☐	ChIP-seq
☒	☐	Flow cytometry
☒	☐	MRI-based neuroimaging

Antibodies

Antibodies used	Polyclonal antisera to the purified, mature bacteroidetocin A and B peptides were raised in rabbits by Lampire Biological Laboratories (Pipersville, PA) using the ExpressLine Basic protocol and were used at a dilution of 1:20. Alkaline phosphatase conjugated goat-anti rabbit IgG (heavy & light chains) secondary antibodies were purchased from Thermo Fisher Scientific (product no. 31340, lot no. QJ226902) and used at a dilution of 1:2000.
Validation	Primary polyclonal and secondary conjugated antibodies were validated by testing their reactivity profiles against appropriate controls.

Animals and other organisms

Policy information about studies involving animals; ARRIVE guidelines recommended for reporting animal research

Laboratory animals	Six female and six male Swiss Webster germ free mice 8 weeks old were obtained from and maintained in the Harvard Digestive Disease Center gnotobiotic core facility at Brigham and Women's Hospital, Boston, MA. Groups of three sex-matched mice were housed in sterile OptiMICE cages (Animal Care Systems, Centennial, CO).
Wild animals	No wild animals were used in this study.
Field-collected samples	No field-collected samples were used in this study.
Ethics oversight	All applicable research described in this paper, including recombinant DNA work and animal studies, was performed in adherence to all ethical regulations, and was reviewed and approved by the Partners Institutional Biosafety Committee (PIBC), Partners HealthCare System, Boston, MA, and/or the Institutional Animal Care and Use Committee (IACUC), Brigham & Women's Hospital, Boston, MA.

Note that full information on the approval of the study protocol must also be provided in the manuscript.
